# Identification of diffusion, kurtosis, and propagator MRI markers of Alzheimer’s disease pathology in post-mortem human tissue

**DOI:** 10.1162/imag_a_00164

**Published:** 2024-05-08

**Authors:** Courtney J. Comrie, Rhea Carlson, Zarif Ahsan, Ashley Moshkriz, Travis W. Sawyer, Anthony J. Intorcia, Geidy E. Serrano, Thomas G. Beach, Elizabeth B. Hutchinson

**Affiliations:** Department of Biomedical Engineering, University of Arizona, Tucson, AZ, United States; Wyant College of Optical Sciences, University of Arizona, Tucson, AZ, United States; Banner Sun Health Research Institute, Sun City, AZ, United States

**Keywords:** microstructural MRI, diffusion MRI microscopy, Braak score, anisotropy, restriction, Alzheimer’s disease

## Abstract

Alzheimer’s disease (AD) is an irreversible degenerative brain disease affecting 6.7 million Americans and while the hallmark AD pathologies of plaques and tangles follow a stereotyped progression during the course of the disease, clinical markers for early diagnosis are lacking and approximately 20% of all AD cases are ultimately misdiagnosed. Conventional clinical MRI is capable of reporting severe brain atrophy, but fails to recognize earlier biomarkers associated with more subtle cellular and molecular changes. Microstructural Magnetic Resonance Imaging (MRI) techniques are promising to address this challenge and may sensitively detect and distinguish tissue degeneration, tauopathies, and beta amyloid plaques to improve accuracy of diagnosis and enable early detection. The objective of this study was to identify and compare the most promising microstructural markers of AD pathology over a range of diffusion and relaxometry-based MRI techniques from conventional to advanced. To accomplish this, we performed MRI microscopy of post-mortem human temporal lobe specimens (n = 14) at high resolution and image quality and evaluated the relative influence of metrics across multiple microstructural MRI frameworks using principal component analysis (PCA). We performed additional correlation analysis between metrics identified by PCA and clinical neuropathology scores of Braak stage and plaque and tangle load. Hippocampal diffusion and restriction metrics contributed most to the first principal component, and the correlation with Braak score was positive for diffusivity and negative for restriction metrics. Additionally, the MAP-MRI propagator anisotropy (PA) metric of microscale anisotropy was strongly and negatively correlated with AD pathology while the conventional fractional anisotropy (FA) metric showed little or no correspondence and there was not a strong association between FA and PA by PCA. Entorhinal cortex findings were minimal except for reported increases in restriction due to plaque content. Taken together, our findings suggest that microstructural MRI metrics of restriction and diffusion are most prominent and may reflect degenerative processes in AD brain tissue and that microscale anisotropy may be more advantageous than conventional FA for the detection of subtle and earlier cellular changes in AD.

## 1 Introduction

Alzheimer’s disease (AD) is an irreversible neurodegenerative brain disease that causes dementia in 6.7 million Americans annually and has become the 5th leading cause of death in the United States for adults over 65 in the year 2023 (Alzheimer’s Association Report, 2023). Given the increasing prevalence of AD and related dementias and the critical need for preventative pharmaceutical or behavioral intervention, there is an urgency to develop accurate early diagnosis methods. These methods should not only detect AD pathology but also distinguish it from common comorbidities. While neuroimaging can potentially serve this purpose, existing clinical tools are limited. For example, conventional magnetic resonance imaging (MRI) methods are commonly used to detect brain atrophy, but MRI is not yet used to detect pathologic changes that appear years before neurodegeneration and clinical symptoms. Currently, Positron Emission Tomography (PET) using Tau and amyloid-β (Aβ) plaque specific radioligands is available for determining threshold pathologic loads of each type of pathology (Mason et al., 2013;Okamura et al., 2014). However, this remarkable capability is limited to proteinopathy specific alterations that do not always correspond with clinical outcomes (Marcus et al., 2014) and cannot assess comorbid pathologies such as Lewy Body Dementia, gross and micro infarcts, microhemorrhages, TDP-43 proteinopathy, and hippocampal sclerosis that are known from pathologic studies to affect 64%–80% brains with confirmed AD (Beach & Malek-Ahmadi, 2021).

Hallmark neuropathologic outcomes observeable only post-mortem have long been used to define AD (Alzheimer et al., 1995;Braak & Braak, 1991b,^1995^;Braak et al., 2006) and if non-invasive, in-vivo markers—by blood or neuroimaging or other approaches—can be developed that measure the presence and stage of these cellular and molecular changes, then diagnostic methods during life and at early stages will be improved. In particular, Braak scoring is used to stage AD upon autopsy by scoring of neurofibrillary tangle (NFT) invasiveness within the brain, which proceeds in a neuroanatomically defined sequence from transentorhinal brain regions (stages I and II), limbic regions especially the hippocampus (stages III and IV) to the eventually extensive neocortical presence of NFTs (stages V and VI). In addition to NFT distribution defined by the Braak stages, AD brain tissue also exhibits Aβ plaques, which follow a spatiotemporal course that is distinct from NFTs. Early stage Aβ plaques first form in the neocortical regions before tangle infestation, and progress to occupy the limbic system, diencephalic regions, brainstem, and cerebellum (Ferrari & Sorbi, 2021;Thal et al., 2002). Importantly, the hallmark pathologies of NFTs and Aβ plaques appear amid concurrent and related degenerative processes of AD, including inflammation, cell loss, and other alterations that greatly influence the local tissue environment.

Microstructural MRI methods that are sensitized to tissue features on the cell and molecular scale by either diffusion (Alexander et al., 2019;Basser & Pierpaoli, 1996;Beaulieu, 2002;Dyrby et al., 2018) or relaxometry (Does, 2018;Sled, 2018) by acquisition and modeling are highly promising for the detection of AD-related tissue and cellular alterations which are robust, prominent, and stereotyped in this disorder. For instance, if the accumulation of NFTs or Aβ plaques considerably alters the concentration of macromolecules within a voxel, then MR relaxivity may be influenced by the presence of proteinopathy. While attempts to use T1 and T2 relaxometry for this purpose have been largely unsuccessful in humans (Tang et al., 2018), more sophisticated acquisition and modeling methods may improve relaxometry-based markers of pathologic macromolecular fractions (Deoni, 2010;Does, 2018). In particular, the use of quantitative magnetization transfer (qMT) pre-pulses to selectively probe water in the hydration layer of large macromolecules and estimate bound pool fraction (BPF) may be influenced by an increase in the density or size of macromolecules due to proteinopathy in AD. Additionally, the separation of short and long T2 components can be accomplished with multi-echo acquisition techniques and compartmental modeling to report relative protein fractions such as myelin water fraction (MWF) (MacKay & Laule, 2016), which may change in both white and gray matter with the progression of AD.

Diffusion MRI (dMRI) frameworks may provide the greatest microstructural sensitivity to alterations of tissue composition and cellular morphology given their unique ability to probe the size and shape features of the microscale tissue environment. Notably, diffusion tensor imaging (DTI) studies have shown increased apparent diffusivity of water associated with degeneration of tissue in the later stages of AD (Goveas et al., 2015;Hong et al., 2010;Ryu et al., 2017) and there is evidence that anisotropy is reduced in AD and healthy aging (Goveas et al., 2015;Ryu et al., 2017;Solar et al., 2021). While such studies suggest that DTI can be sensitive to AD pathology, especially at later stages, the specificity of DTI metrics with particular pathologic underpinnings is unclear and more sophisticated dMRI methods that may increase both sensitivity and specificity are only beginning to be studied (Avram et al., 2016;Frank et al., 2020). Among the most promising dMRI methods are non-Gaussian techniques, including diffusion kurtosis imaging (DKI) (Jensen et al., 2005) and mean apparent propagator MRI (MAP-MRI) (Ozarslan et al., 2013) that are sensitive to changes in restricted water compartments, which may be affected by AD pathology. MAP-MRI additionally provides an estimate of microscale anisotropy by the propagator anisotropy (PA) metric, which unlike DTI fractional anisotropy (FA) is less influenced by the architectural organization of tissue features. This improved selectivity should be advantageous for detecting subtle alterations in microscale features (e.g., neurite shape or density, fiber shapes in regions of crossing) that may be altered in AD prior to degeneration.

The potential for developing new microstructural MRI markers in AD is limited by the lack of “ground truth” validation for AD pathology and comorbidities in living patients as these cannot be known before post-mortem neuropathology assessment. In order to establish meaningful radiologic-pathologic correspondence between microstructural MRI techniques, it is essential to draw direct comparisons between quantitative MRI values and the presence and load of NFTs, Aβ plaques, and comorbid pathology. In the present study, MRI microscopy of post-mortem tissue specimens from donors with a range of Braak stage, NFT, and Aβ pathology was collected to generate high-resolution and high-quality microstructural MRI maps, including DTI, DKI, and MAP-MRI metrics as well as T1, T2, BPF, and MWF. The primary objective of this study is to identify the most promising microstructural MRI markers within the post-mortem dataset, with the overarching aim of informing future development of in-vivo imaging tools. Principal component analysis was used to determine relative influence and associations across metrics and secondary; descriptive correlation analyses were performed with quantitative neuropathology scores in the hippocampus and entorhinal cortex to relate these microstructural MRI techniques with AD pathology.

## 2 MRI Methods

### 2.1 Clinical characteristics and neuropathology

All specimens were acquired with informed consent during life by adults under the Common Rule regulations and their health information is protected by the Health Insurance Portability and Accountability Act of 1996 (HIPPA) (Beach et al., 2015). Fourteen temporal lobe specimens were received and prepared by the Arizona Study of Aging and Neurodegenerative Disorders/Banner Sun Health Brain and Body Donation program according to established autopsy and post-mortem tissue processing methods (Beach et al., 2015). For all 14 specimens, Braak staging, Aβ-plaque, and tau-tangle scoring were performed by an experienced neuropathologist using established protocols with sections taken from the block prior to MRI and from other regions of the brain (as required for Braak staging). Plaque and tangle densities in the CA1 hippocampus subfield, stained with the Campbell-Switzer and Gallyas silver stainsFigure 1, respectively, were assigned a semi-quantitative score of none, sparse, moderate, or frequent (converted to 0–3 for quantitative purposes) (Braak & Braak, 1991a). Demographic, clinical, and comorbidity details of the specimen donors are given inTable 1along with Alzheimer’s pathology scores. Comorbidities aside from AD, which were examined during autopsy to determine their presence or absence within our dataset, include the following conditions: vascular dementia (VAD), progressive supranuclear palsy (PSP), hippocampal sclerosis (HS), dementia lacking distinctive histology (DLDH), motor neuron disease (MND), corticobasal degeneration (CBD), Pick’s disease, Huntington’s disease (HD), multiple system atrophy (MSA), frontotemporal lobar dementia with TDP-43 proteinopathy (FTLD-TDP), cerebral white matter rarefaction (CWMR), and Lewy bodies (LBS) (note: while LBS were present in some specimens, none met the diagnostic criteria for dementia with Lewy bodies or Parkinson’s disease) (Cairns et al., 2007;Dickson, 2005;Dickson et al., 2009). Subject age at time of autopsy is also listed inTable 1and it should be noted that there is not a positive correlation between age and pathologic score but rather modest negative correlation [ρ_Braak, Plaques, Tangles_= -0.57, -0.32, -0.56] that is expected given the fatal outcome of AD. Two representative samples—one with AD diagnosis during life and one without—were selected for initial protocol development and optimization and unblinded qualitative assessment. The remaining 12 samples were imaged and quantitatively analyzed in a blinded manner without knowledge of clinical or pathologic status by the imaging team.

**Fig. 1.**
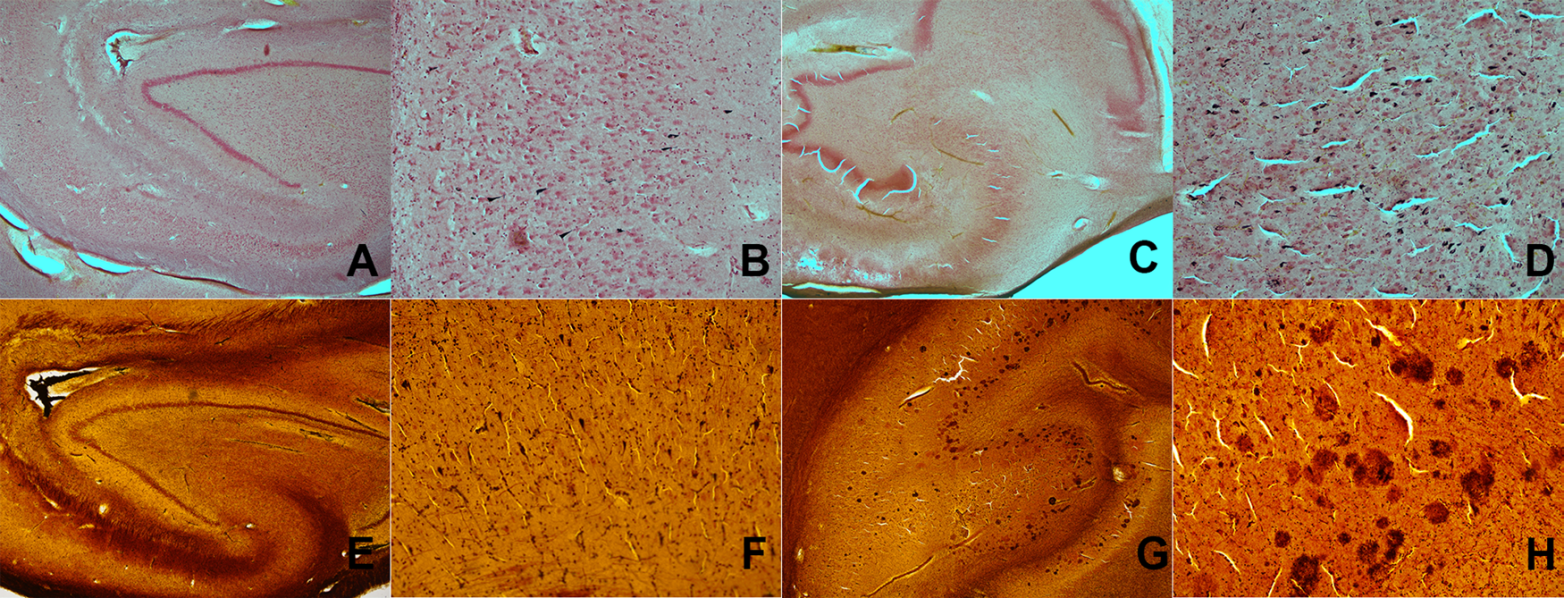
Histochemical staining of representative samples for pathological scoring. Gallyas staining (top row) was used to show neurofibril tangles, and Campbell-Switzer staining (bottom row) showed plaques. Two magnifications (2× and 4× left to right) are shown for the healthy (A, B, E, and F) and the diseased (C, D, G, and H).

**Table 1.** Demographic and neuropathology data for 14 post-mortem samples.

Dataset	ID	BRAAK score	HIP tangles	HIP plaque	EC tangles	EC plaque	Age	Sex	Comorbid pathology
Representative	1	VI	3	2	3	3	71	M	ARG, CWMR, LBS
	2	IV	2	0	2.5	0	88	M	None
12 Sample	3	IV	3	1	2.75	2.5	83	M	LBS
	4	II	0.5	1.5	1.5	1.5	87	M	CWMR
	5	VI	3	3	3	3	85	M	None
	6	III	1.5	0	1.5	0	86	M	ARG, CWMR
	7	VI	3	1.5	3	3	75	M	None
	8	VI	3	3	3	3	85	F	CWMR
	9	VI	3	3	3	3	50	F	CWMR
	10	IV	2	1.5	3	2.5	89	M	None
	11	V	2	2	3	2	89	M	ARG, CWMR
	12	V	3	2.5	3	3	84	M	PSP, ARG
	13	VI	3	1.5	3	3	79	F	None
	14	III	1	3	2	3	82	M	VAD

The columns are as follows: sample ID, Braak score, hippocampus (HIP) tangle count, hip plaque count, entorhinal cortex (EC) tangle count, and EC plaque count, age at time of death, gender, and comorbidities. Only the Braak score, hip tangles, hip plaque, EC tangles, and EC plaques categories were used for analysis. Comorbidities acronyms are as follows: argyrophilic grains (ARG), cerebral white matter rarefaction (CWMR), lewy bodies (LBS) progressive supranuclear palsy (PSP), and vascular dementia (VAD).

### 2.2 Sample preparation

According to standard, well-established practices of the Banner Brain and Body Donation Program (Beach et al., 2015), 1 cm coronal blocks were prepared immediately after brain extraction. These blocks then underwent immersion fixation in commercial formalin containing 4% formaldehyde for 2 days followed by storage in phosphate buffered saline with 0.1% sodium azide. This consistent preparation procedure combined with a short post-mortem interval provided a critical reduction in bias from known degradation, fixation, and re-hydration effects (Shepherd et al., 2009). Sections were taken from the temporal lobe block and processed for the neuropathologic stains as described in the previous section, and the remaining tissue block was sent to the imaging team. Prior to MRI, samples were additionally trimmed to fit the sample holder and MRI coil to a maximum diameter of 2.5 cm with care to include the hippocampus and entorhinal cortex (EC). On the day of imaging, multiple (2–3) specimens were selected and placed together in a 50 mL Falcon tube containing the non-protonated fluid fluorinert (FC-3823, 3M, St. Paul, MN). Gauze and plastic spacers were used to stabilize and separate the specimens within the tube, and air bubbles were removed using a vacuum chamber.

### 2.3 MRI acquisition and processing

All MRI scans were collected using a Bruker BioSpec 70/20 USR 7T MRI with the Paravision 360 2.0 software package. The following microstructural MRI scans were acquired and processed as described below and inFigure 2.

**Fig. 2.**
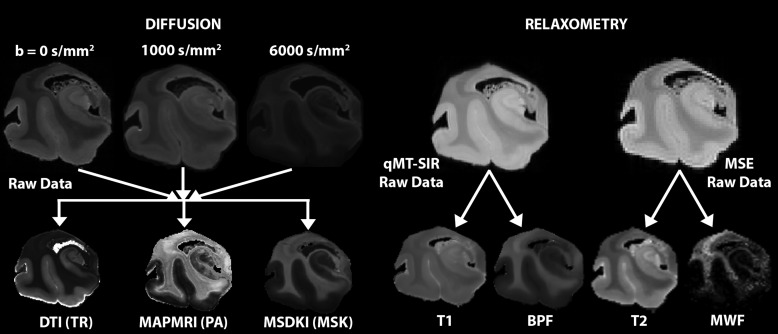
Image processing pipelines for diffusion (left) and relaxometry (right) microstructural MRI techniques. Unprocessed diffusion weighted images (DWIs) with different b-values are shown and resulting scalar output maps for diffusion tensor imaging (DTI) Trace (TR), mean apparent propagator (MAPMRI) propagator anisotropy (PA), and diffusion kurtosis imaging (DKI) mean signal kurtosis (MSK) are shown. Unprocessed relaxometry data for both quantitative magnetization transfer selective inversion recovery (qMT-SIR) and multi-spin echo (MSE) are shown and maps generated by REMMI processing include T1, bound pool fraction (BPF), T2, and myelin water fraction (MWF).

#### 2.3.1 Diffusion MRI

Diffusion weighted image volumes were collected using a 3D, spin-echo EPI pulse sequence with TE/TR = 50/800 ms, Δ/δ = 20/5 ms, 250-micron isotropic voxel dimensions, NEX = 1, segments = 6, and two repetitions having opposite phase encode direction to enable correction of geometric distortions. Multi-shell diffusion encoding was b(# directions) = 0, 100(6), 500(6), 1000(6), 1500(30), 3000(30), 4500(60), and 6000(60) for a total of 201 DWIs. Diffusion data were denoised (Veraart et al., 2016) and corrected for Gibbs ringing (Kellner et al., 2016), apparent motion, and distortion artifacts using TORTOISE version 3.2.0 (Irfanoglu, 2018). Four diffusion models were applied to the DWIs: DTI (Basser et al., 1994) to generate Trace (TR) and FA maps, mean signal diffusion kurtosis imaging (MSDKI) (Henriques et al., 2021;Jensen et al., 2005) to generate mean signal diffusivity (MSD) and mean signal kurtosis (MSK) maps, which report unbiased diffusivity and restriction respectively, and MAP-MRI (Ozarslan et al., 2013) to generate return to origin probability (RTOP) and PA maps, which report isotropic restriction and microscale anisotropy respectively. DTI and MAP-MRI fitting and metric map generation were accomplished using the TORTOISE software package (Irfanoglu, 2018), and the DKI model was applied via the DIPY Python library.

#### 2.3.2 T1-relaxometry and BPF mapping

Quantitative magnetization transfer Selective Inversion Recovery (qmt-SIR) was implemented using a 3D RARE pulse sequence with: TE/TR = 6/1617 ms, IR = [6, 9.1, 13.8, 20.8, 31.5, 47.8, 72.3, 109.5, 165.9, 251.2, 380.4, 576, 872.2, 1320.8, and 2000] ms, RARE factor of 16, and voxel dimensions of approximately 250 microns isotropic. The T1 and BPF were mapped using the REMMI software (Harkins, 2016).

#### 2.3.3 T2-relaxometry and MWF mapping

Multi-echo, spin-echo MRI volumes were collected using a 3D RARE pulse sequence with 32 different echo time from 6.5–208 ms with an echo spacing of 6.5 ms, TR = 1000 ms, and voxel dimensions of 250 isotropic microns. A high-resolution anatomical (HRA) image was generated by averaging all weighted images, and this HRA was used in later steps for within-subject registration and region of interest drawing. For quantitative mapping, the T2 and MWF were calculated using the REMMI software package (Harkins, 2016).

### 2.4 Dimension reduction and data visualization

**Fig. 3.**
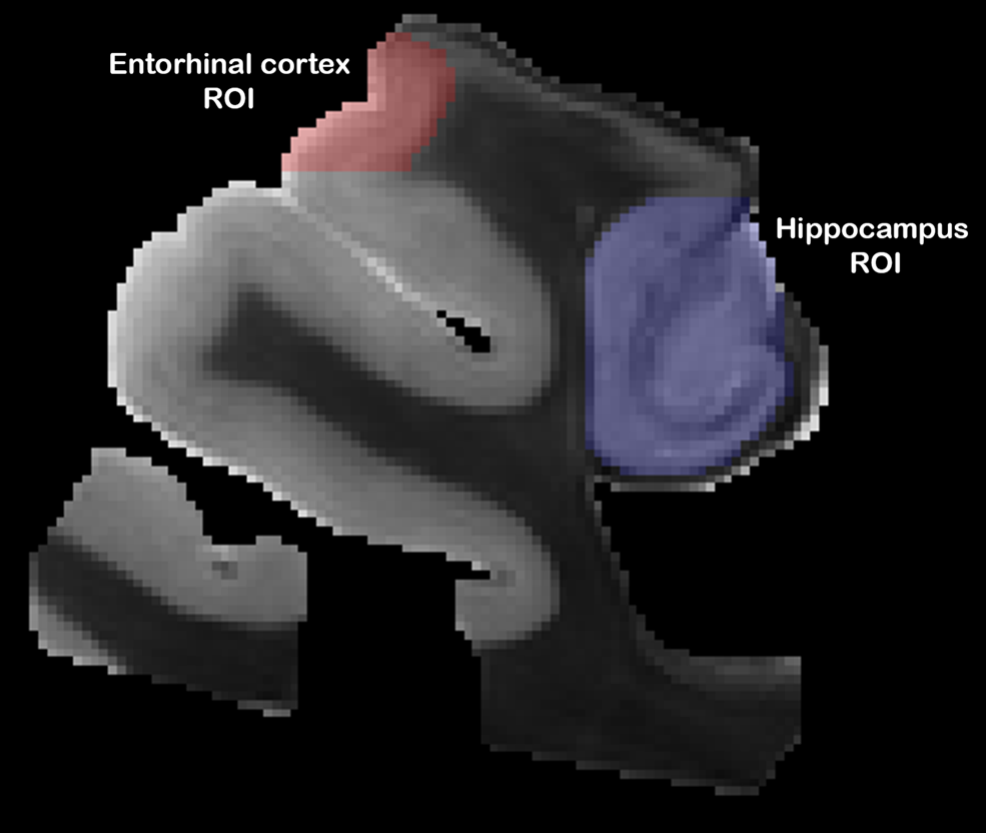
Visualization of manually drawn hippocampal (blue) and entorhinal cortex (red) ROIs on specimen 6.

While the tissue blocks were prepared from consistent anatomical location in each brain, individual differences in the size, shape, and slice orientation across specimens precluded cross-sample registration or voxel-wise analysis and region of interest (ROI)-based analysis was performed. For each specimen, all microstructural MRI maps were rigidly registered to the HRA image space using TORTOISE or ANTS (Avants, 2015) software. Region-of-interest masks (Fig. 3) were created manually for the EC and the hippocampus using the ITKsnap software (Yushkevich et al., 2006). An in house, consistent, manual segmentation approach was taken for both anatomical regions. Briefly, hippocampal ROIs were drawn in a coronal view aiming to capture CA1 through CA4/dente gyrus (also including perforant pathway). The EC was identified by first locating the subiculum and perirhinal cortex and using these two landmarks to define the extent of the ROI. Consistent ROI placement for both regions was confirmed in the other orthogonal views, and accuracy of ROI segmentation for these regions was confirmed by discussion with a neuropathologist. Two specimens, numbers 3 and 13 (Table 1), were excluded for the entorhinal cortex region and one mask, number 3, was excluded for the hippocampus due to inadequate anatomy present on MRI scans. Voxel values for each metric map were extracted using the ROI mask, and individual histograms were created for each specimen using Python. Mean values were also calculated for each metric and ROI of each specimen.

Initial descriptive correlation matrices were generated for the hippocampus and EC separately using the DataFrame.corr command in the python pandas library to automatically calculate Spearman’s correlation coefficient from mean MRI metric values and quantitative pathological scores (Supplemental Figs. 1 and 2). After the initial correlation analysis highlighted metric relationships, principal component analysis (PCA) was done to more rigorously inspect the metric relationships. The correlation matrices were reduced to include only the MRI metric correlations and used as inputs to PCA. PCA was performed using the decomposition. PCA command within the sklearn python library with 13 PCs, and explained variance and component loadings are visualized using scree and biplots respectively (Jolliffe & Cadima, 2016). To indicate metric relationships with pathology, arrow colors on the biplots were mapped as Spearman’s ρ value for each metric correlation with Braak Score. For group comparisons, normalized and averaged histograms were created from all individual histograms from samples with similar Braak score according to low (stages I-III), medium (stages IV-V), and high (stage VI) in Matlab (R2021b, Natick, MA). Finally, scatter plots were generated between metrics of interest and Braak score using the Python command scatter in the matplotlib library along with a bootstrapping random analysis performed for Spearman’s ρ values with 1000 iterations to provide a [5, 95] confidence interval (CI) for the displayed metric and Braak Score Spearman’s ρ values. The CIs were used to inform the interpretation of ρ values as indicating strong correlation when the full range of the CI did not include zero.

### 2.5 Histology and OrientationJ mapping

Following MRI, both representative specimens were processed for additional histopathology (beyond diagnostic staining which was performed for all 14 samples). For immunohistochemistry, paraffin-embedded blocks collected adjacent to the blocks used for imaged were sectioned at 6 µm. Sections were stained with primary antibodies against p-tau (clone AT8, Thermo Scientist), Aβ (6E10, Covance), astrocytes (GFAP, Millipore), and microglia (IBA, Wako). The immunohistochemical methods were identical except for the usage of differing epitope exposure methods: 20 min in 80% formic acid for p-tau, Aβ, and GFAP and 30 min in boiling 1 mM EDTA (pH 9) for IBA1. White matter integrity was assessed by immunohistochemistry using myelin basic protein (myelin, LSBio) histological protocol (Serrano et al., 2015). Slides were imaged using a Nikon BioPipeline Slide Scanner at 10x objective and resolution of 0.34 mm/pixel. In addition to IHC processing and microscopy, the membrane staining approach of 1,1'-Dioctadecyl-3,3,3',3'-Tetramethylindocarbocyanine Perchlorate (DiI) was used with structure tensor analysis (Budde & Frank, 2012) to assess the orientation and coherency of cellular structures in the hippocampus of the two representative specimens. Slides were submerged in a 100% xylene bath for deparaffinization of tissue (25 min) and immediately rinsed in an ethanol bath to remove any remaining xylene (20 min). Sections were rinsed for 10 sec in 0.25 mg/mL DiI, dissolved in 100% ethanol solution, and rehydrated in a graded ethanol series (95–0%) for 10 min in each bath before coverslipping with Vectashield, antifade mounting media (Budde et al., 2011). DiI slides were imaged on a Nikon Eclipse E600 light microscope using a 4x and 10x objective lens. Using ImageJ, the RBG images obtained in bright field imaging were converted to 8-bit images (Rasband, 1997–2018). The alignment of fibers within the samples was assessed using OrientationJ, an ImageJ plugin (Puspoki et al., 2016). OrientationJ was applied using a structure tensor with a Gaussian window of 2 pixels and a cubic spline gradient algorithm where the hue corresponds to orientation, the color saturation corresponds to the fiber coherency, overlaid onto the 8-bit image (Puspoki et al., 2016).

## 3 Results

**Fig. 4.**
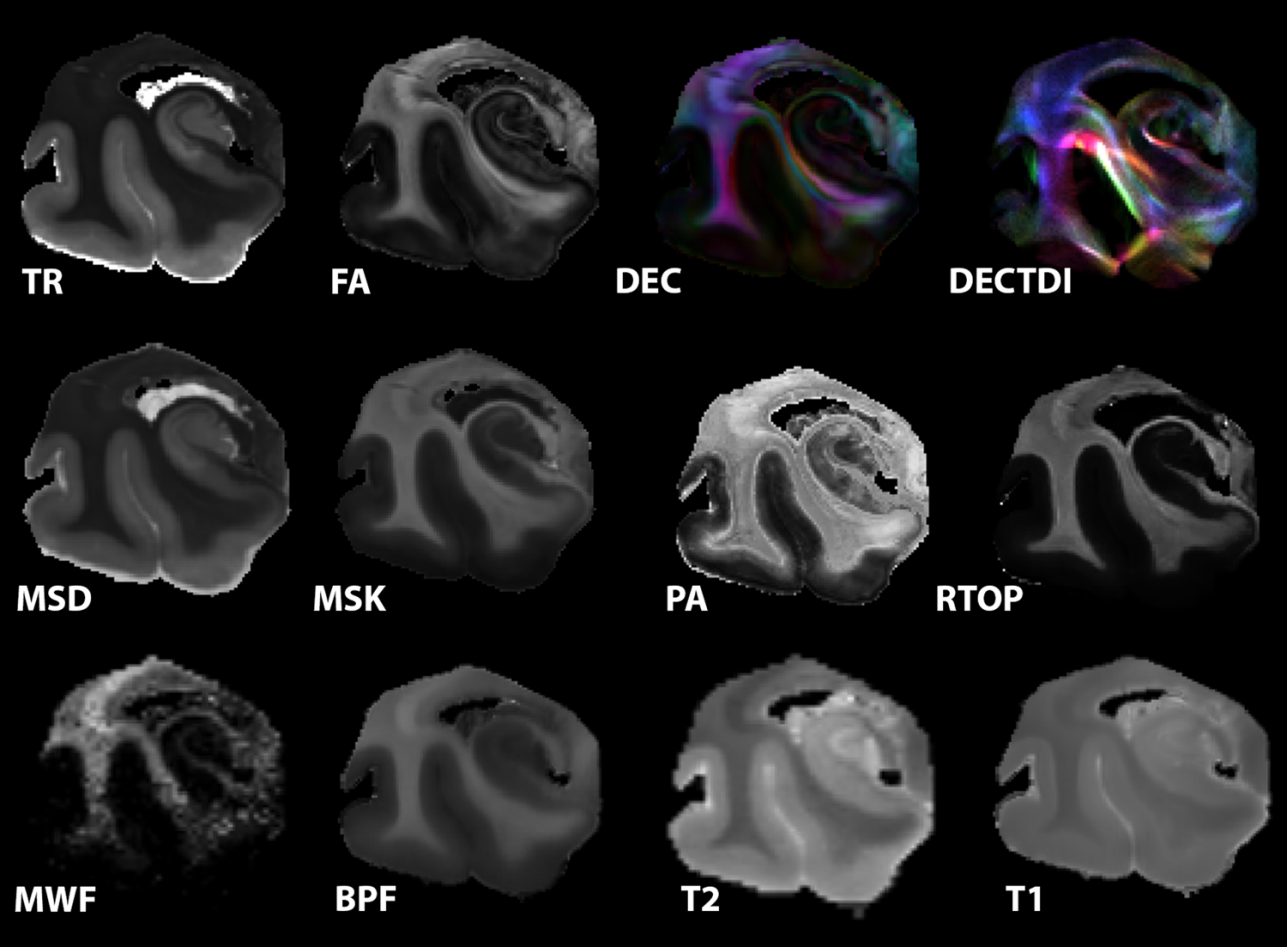
Overview of metric maps investigated in this study. For the same slice in the representative healthy aged specimen, the following maps are shown: trace (TR), fractional anisotropy (FA), diffusion encoded color (DEC), diffusion encoded color tract density image (DECTDI), mean-signal diffusivity (MSD), mean-signal kurtosis (MSK), propagator anisotropy (PA), return to origin probability (RTOP), myelin water fraction (MWF), bound pool fraction (BPF), T2, and T1.

**Fig. 5.**
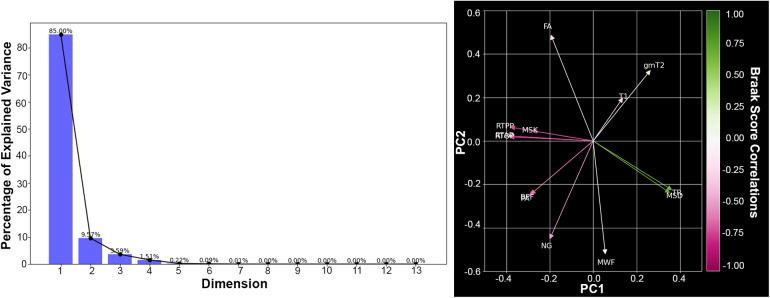
Principal component analysis of microstructural MRI metrics in the hippocampus. The scree plot (left) shows the explained variance (y-axis) for each of the 13 principal components (x-axis). The biplot (right) is a visual representation for each of the loadings effect on the PC1 versus PC2. Where the arrow color is determined by the MR metric’s Spearman’s correlation ρ value with Braak score.

The MRI acquisition and processing pipelines optimized for these post-mortem specimens produced high-resolution and high-quality maps (Fig. 4) in which the subfields and layers of the hippocampus were evident. Explained variance across MRI metrics in the hippocampus was evaluated using PCA and reported graphically (Fig. 5) by scree plot and biplot along with ranked contributions of each metric for PC1 and PC2 (Table 2). PC1 and PC2 explained 94.18% of the variance. Metrics of diffusivity (TR and MSD) were grouped together in the biplot with high ranking in PC1 and were positively correlated with Braak score (green color mapping). Metrics related to restriction (MSK, RTOP, RTPP, RTAP) were grouped together and opposite in direction to the diffusivity metrics on the biplot and were negatively correlated with Braak score (magenta color mapping). Relaxivity metrics of T1 and gmT2 were in the same direction on the biplot, but had only modest ranking in either PC and no strong correlation with Braak score. PC2 was influenced the most by FA, MWF, and NG although these metrics were not strongly grouped and did have high correlation with Braak score. Finally, BPF and PA were grouped together on the biplot with modest influence on both PC1 and PC2 and negative correlation with Braak score.

**Table 2.** Principal component loadings for principal component 1 (PC1) (middle column) and PC2 (right column) for the MRI metrics (left column) in the hippocampus.

Metric	PC1	PC2
RTAP	-0.3651	0.022016
RTOP	-0.3638	0.017768
RTPP	-0.36163	0.060192
TR	0.344894	-0.21446
MSD	0.33582	-0.2273
PA	-0.27897	-0.23846
BPF	-0.27147	-0.2322
MSK	-0.25949	0.043518
gmT2	0.250742	0.309357
NG	-0.19148	-0.43209
FA	-0.18715	0.468599
T1	0.124692	0.181094
MWF	0.053724	-0.50046

The table is organized in descending order based on metrics impact on PC1.

**Fig. 6.**
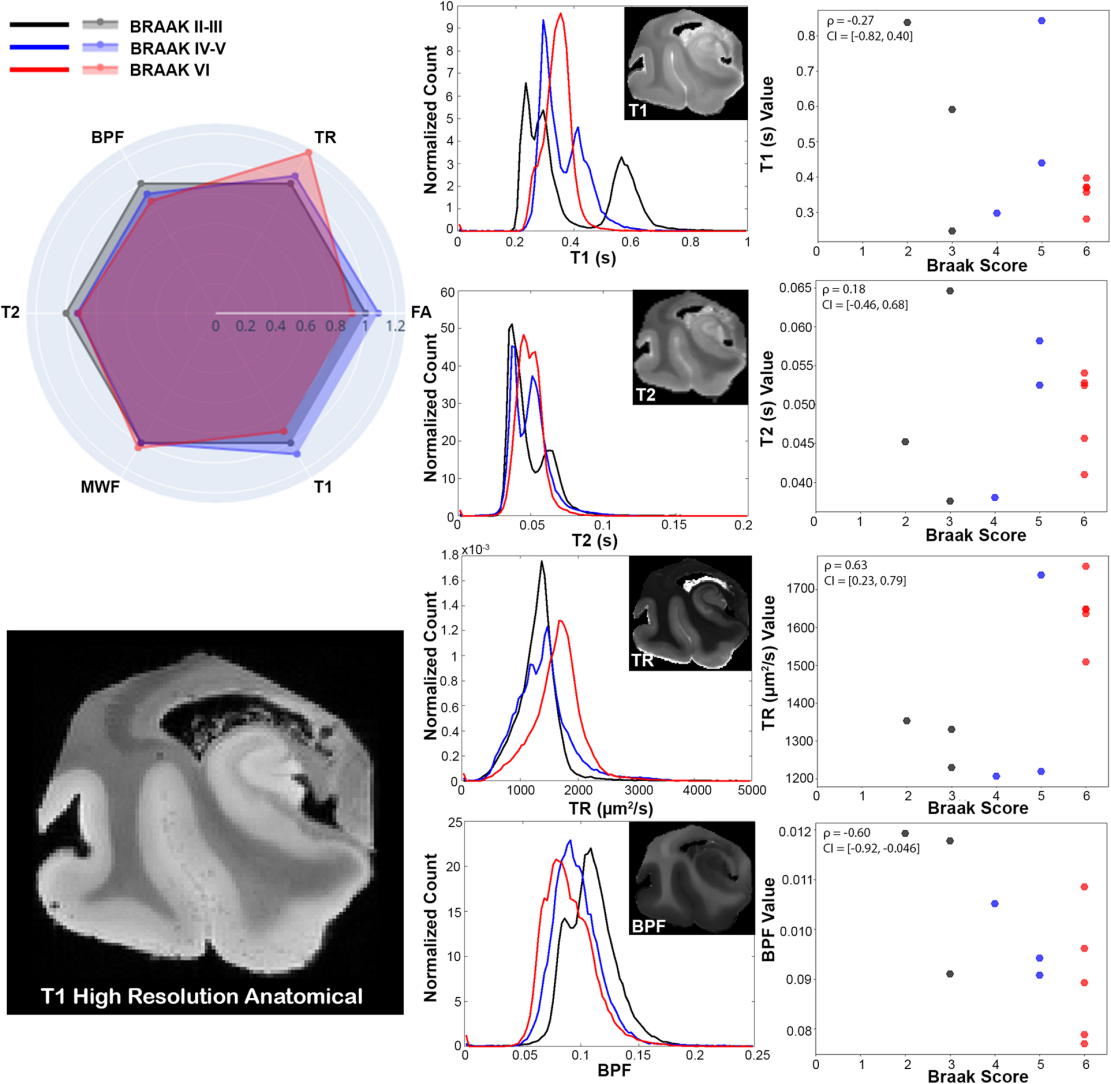
Quantitative analysis in the hippocampus of microstructural relaxometry and DTI metrics according to Braak stage. Radar chart (top left) shows averaged and normalized metric values across Braak stage groups. Averaged normalized histograms (middle) across Braak staged groups, and scatter plots showing average metric values in hippocampal ROIs over corresponding specimen Braak score. Representative images for characteristic healthy sample are displayed for reference with structural T1 High Resolution Anatomical, T2 map, bound pool fraction, and trace image. Where the black indicates Braak staged groups II-III, blue is IV-V, and red is VI.

**Fig. 7.**
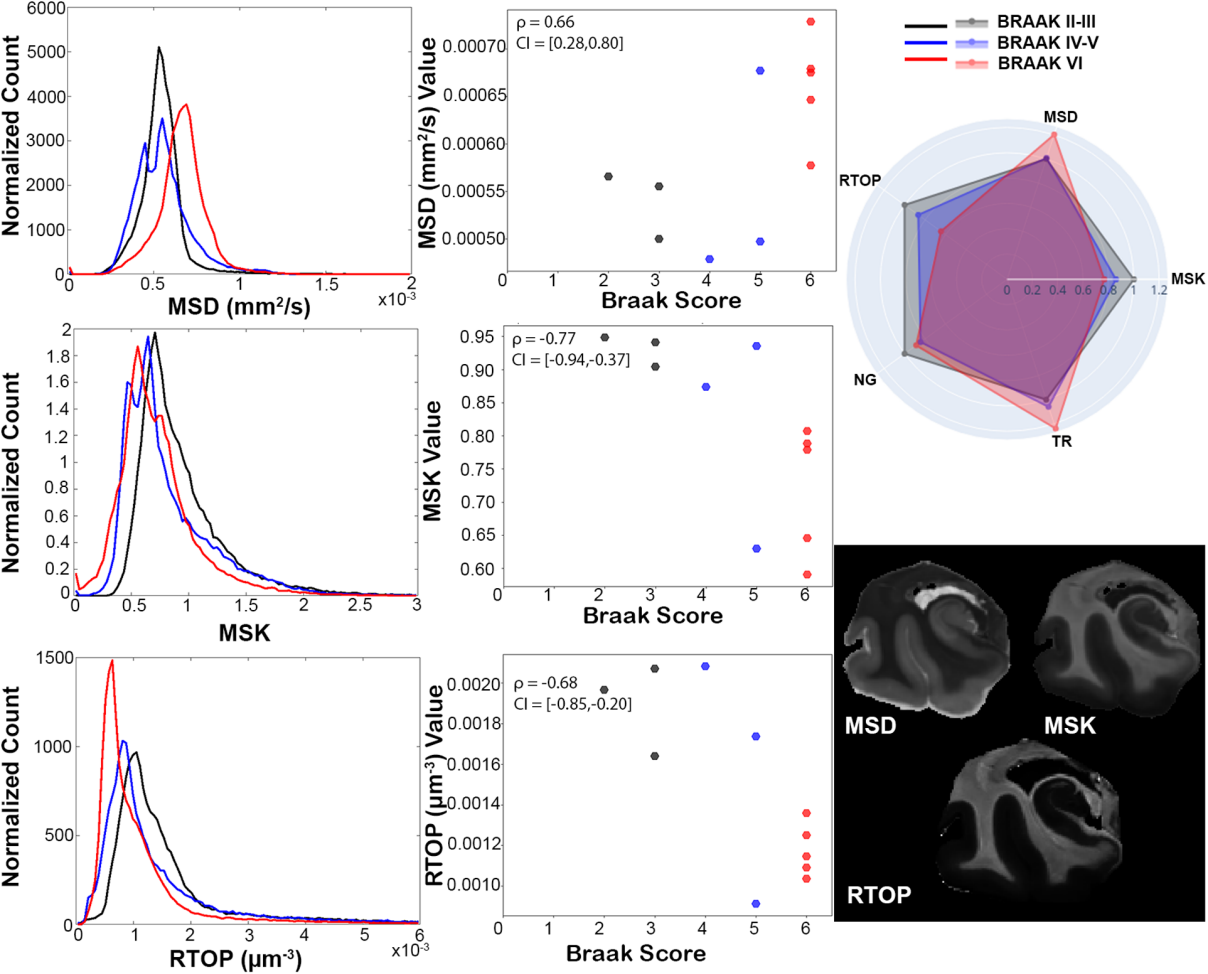
Quantitative analysis in the hippocampus of restriction and diffusion metrics according to Braak stage. Radar chart (top right) shows averaged and normalized metric values across Braak stage groups. Averaged normalized histograms across Braak stage groups (right) and scatter plots of average metric values in hippocampus ROIs over corresponding Braak stages. Representative images for characteristic healthy sample (bottom right) are displayed for reference with mean signal diffusivity (MSD), mean signal kurtosis (MSK), and return to origin probability (RTOP) image. Where the black indicates Braak staged groups II-III, blue is IV-V, and red is VI.

Additional descriptive analysis and statistics were performed using histograms, scatter plots, and Spearman’s correlation. Although the relatively low sample size and large number of metrics in this study preclude inferential statistical analysis due to low power and multiple comparisons concerns, clear relationships for some metrics were evident by eye in scatter plots and supported strongly by Spearman’s correlation bootstrapped confidence intervals, which for some metrics were fully exclusive of zero correlation. For these metrics, the abnormalities were also prominent by the eye in the metric maps of the temporal lobe. Braak score was positively correlated with diffusivity-related metrics of TR and MSD (ρ_TR_= 0.63 and ρ_MSD_= 0.66,Figs. 6and7) and negatively correlated with restriction-related metrics of MSK and RTOP (ρ_MSK_= -0.77 and ρ_RTOP_= -0.68,Fig. 7). There was a negative correlation with BPF (ρ_BPF_= -0.6) and microscale anisotropy by PA (ρ_PA_= -0.69) but not with FA (ρ_FA_= -0.24) or MWF (ρ_MWF_= 0.19).

The additional pathology scores of hippocampal plaque and tangle counts showed similar trends (seeSupplemental Material) with the exception of a divergence in T2 correlation with plaques but not tangles (Plaques: ρ_gmT2_= 0.48; Tangles: ρ_gmT2_= 0.099) and a converse pattern for BPF and MSK, which had a stronger negative correlation with tangles than plaques (Plaques: ρ_BPF_= -0.26, ρ_MSK_= -0.21; Tangles: ρ_BPF_= -0.53, ρ_MSK_= -0.62). The two anisotropy metrics showed divergent correlations with pathology score with FA more strongly, negatively correlated with plaques and PA more strongly negatively correlated with tangles (Plaques: ρ_FA_= -0.48, ρ_PA_= -0.25; Tangles: ρ_FA_= -0.06, ρ_PA_= -0.63).

**Fig. 8:**
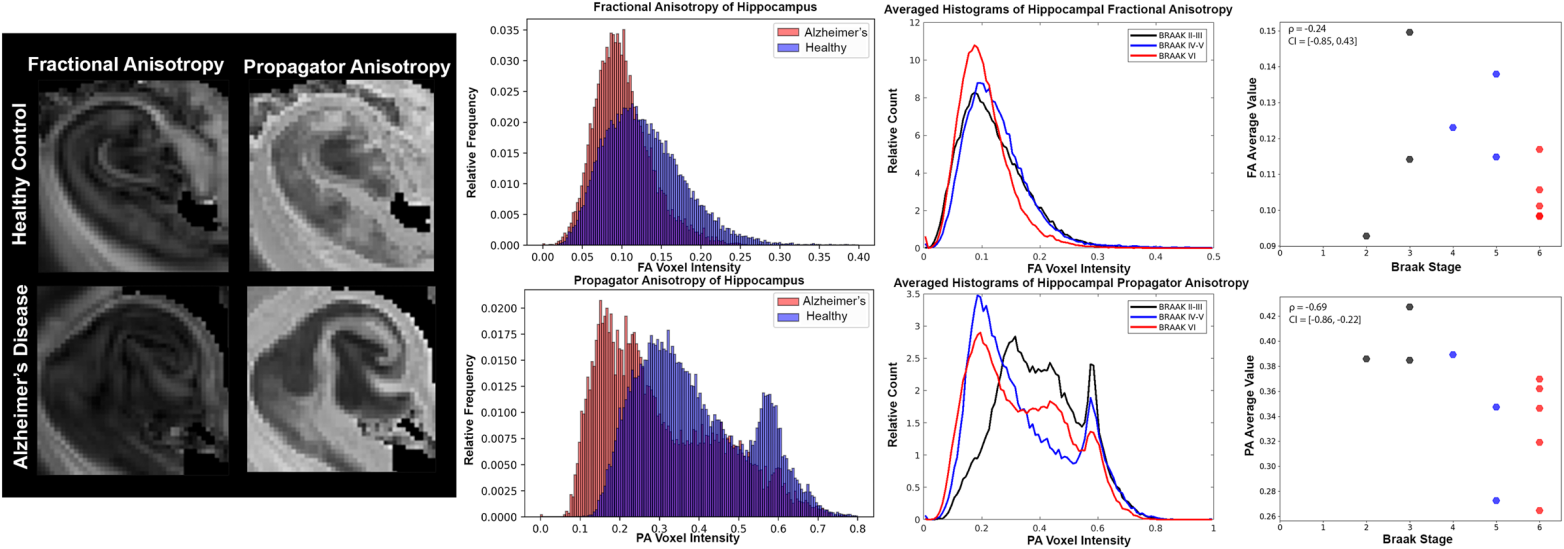
Quantitative analysis of fractional and propagator anisotropy according to Braak stage. Magnified fractional anisotropy (FA) and propagator anisotropy (PA) maps in the region of the hippocampus (left). Histogram analysis of FA and PA values between representative AD (red) and healthy (blue) specimens (middle left). Average normalized histograms across Braak stage groups (middle right), and scatter plots of averaged metric values by specimen Braak where black is Braak scores II-III, blue is scores IV-V, and red is VI.

Histogram and scatter plot comparative analyses were performed for DTI and relaxometry techniques (Fig. 6), non-Gaussian diffusion MRI methods (Fig. 7), and anisotropy metrics (Fig. 8). The radar plot inFigure 6shows that the greatest metric differences between the healthy and AD representative specimens are in TR, while the other metrics were not prominently different between the two. This was visually apparent as well within the hippocampus where TR contrast of the hippocampal tissue was remarkably greater in the AD brain unlike for the other metric maps. This is further confirmed by the histogram and scatter plots where clear separation in TR values according to Braak stage is apparent.

**Fig. 9.**
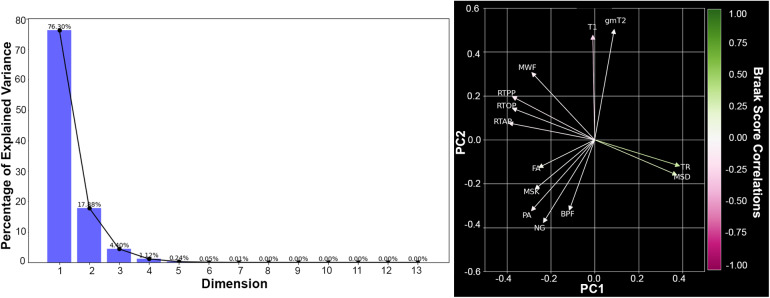
Principal component analysis of microstructural MRI metrics in the entorhinal cortex. The scree plot (left) shows the explained variance (y-axis) for each of the 13 principal components (x-axis). The biplot (right) is a visual representation for each of the loadings effects on the PC1 versus PC2. Where the arrow color is determined by the MR metric’s Spearman’s correlation ρ value with Braak score.

The DKI metric of MSD was found to be similarly increased in the AD specimen as TR on the radar plot and to have similar histogram and scatter distribution patterns across Braak stage groups inFigure 7. The other DKI metric of MSK, which is more related to non-Gaussian restricted water, was decreased in the AD specimen and histograms were shifted toward lower values with increasing Braak score. Additionally, the scatter plots showed reduced restriction at Braak stage VI. While the MAP-MRI metric of non-Gaussianity (NG) was not appreciably different in the AD specimen, RTOP was decreased, and both the histogram and scatter distribution were shifted to lower values and narrower with increasing Braak stage.

**Table 3.** Principal component loadings for principal component 1 (PC1) (middle column) and PC2 (right column) for the MRI metrics (left column) in the entorhinal.

Metric	PC1	PC2
RTAP	-0.37295	0.072639
TR	0.368703	-0.11339
RTPP	-0.35772	0.187629
RTOP	-0.3571	0.136826
MSD	0.356575	-0.15284
PA	-0.27543	-0.30894
MWF	-0.27345	0.292427
MSK	-0.25675	-0.21303
FA	-0.23752	-0.11807
NG	-0.22369	-0.36059
BPF	-0.10914	-0.30293
gmT2	0.085757	0.483522
T1	-0.00805	0.45685

The table is organized in descending order based on metrics impact on PC1.

A comparative analysis of anisotropy metrics is shown inFigure 8. The histogram comparison between AD and healthy hippocampus for FA and PA in the representative samples showed similar distribution shape for FA between AD and healthy tissue with a shift in the AD sample to lower values and a narrower range. The PA histogram distribution was quite distinct from FA in having a prominent bimodal shape, especially in the healthy hippocampus in which the higher PA values were localized to axon-rich regions such as the perforant path and lower PA values were found in the dendrite-rich layers of the hippocampus. Compared with the healthy tissue, the AD distribution showed a diminished higher peak, but no shift in the upper range of values. The lower range of PA values did shift for the AD tissue, including the peak and minimum values. The same histogram features and changes with AD were consistent across the other 11 samples in the hippocampus and PA changes were evident at earlier Braak stages than changes in FA. The low and medium (black and blue) Braak stage groups were similar in FA histograms, while the stage VI (red) histogram profile was decreased in mid-range values. Grouped PA histogram profiles for all 11 samples had a bimodal distribution, and shifts in the histogram from the low Braak score group were observed for both the medium and high Braak stage groups. PA was the only metric for which appreciable changes in the histogram were observed for lower Braak scored groups.

These observations were confirmed by scatter plots with PA showing a decline in anisotropy between low (II-III) and middle (IV-V) Braak stages, while FA did not show a clear pattern or early stage reduction.

PCA analysis of MR metrics within in the EC (Fig. 9,Table 3) showed explained variance predominately captured within the first two PCs (94.18 %), although the biplot groupings were not as tight as for the hippocampus and the correlation values far lower. PC1 was again predominantly influenced by diffusivity and restriction metrics while unlike in the hippocampus, the EC PC2 was more influenced by relaxivity metrics (T1 and gmT2) than by FA, BPF, or MWF. Secondary radiologic-pathologic correspondence analysis in the EC showed a strong positive correlation (ρ_RTOP_= 0.57) between RTOP and Aβ plaque score in the EC. Additionally, FA and MWF were positively correlated with plaque score (ρ_FA_= 0.32, ρ_MWF_= 0.55).

**Fig. 10.**
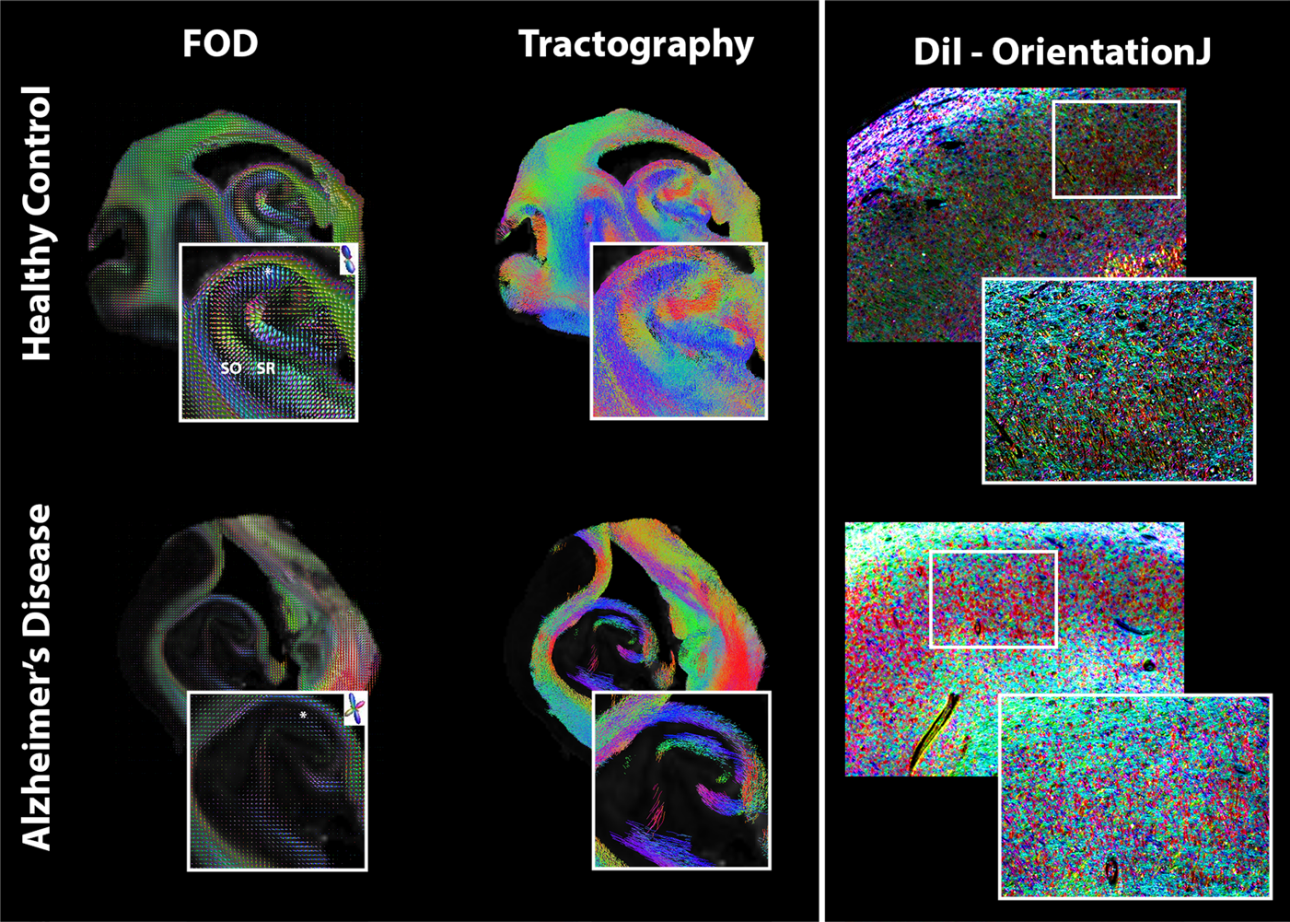
Orientation maps between healthy (top) and Alzheimer’s disease (bottom) representative specimens with MRI maps on the left and quantitative histology maps on the right where orientation dispersion index (ODI) shows clear laminar distinctions between the stratum oriens (SO) and stratum radiatum (SR) that are disrupted in the AD specimen. CSD tractography and fiber orientation distribution (FOD) glyphs are different between the two specimens. A single FOD glyph for each specimen is shown at the CA3 region indicated by *. Histology-based orientation maps using DiI in the CA3 region are shown at 4x and 10x magnification where the white box indicates the 10x region.

In addition to scalar metrics, orientational features of the hippocampal layers were compared with FOD and tractography maps (Fig. 10). For the healthy hippocampus, FOD maps showed remarkable separation of hippocampal layers in the various subfields, including lower dispersion in regions of coherent neurites (e.g., within the stratum radiatum, SR) and higher dispersion in regions of crossing fibers (e.g., in deeper regions of the SR or stratum lacunosum molecularae where axon collaterals cross other neurites) and in regions of disperse neurite orientation (e.g., in stratum oriens, SO). In the AD hippocampus, the demarcation of these layers was unclear. Constrained spherical deconvolution-based (CSD) tractography showed selective alterations in the hippocampal layers for the AD specimen. Specifically, there was a loss of tracts in the locations and orientations of pyramidal cell neurites but preservation of tracts in the locations and orientations of axonal fibers (Fig. 10). DiI stained sections and orientation maps of these revealed decreased microstructural fiber density and complexity in the AD specimens, although the main direction of orientation was similar between the two samples.

Additional histologic staining for both representative samples is shown inFigure 11. The AD specimen clearly shows presence of tangles and plaques (AT8 and 6E10) in the CA1 region of the hippocampus, while the healthy specimen has none. Myelin staining appears similar for the main axonal pathways of the hippocampus, but there is a reduction of myelinated projection fibers in the SO and SR hippocampal layers. Activated microglia (IBA1) are seen throughout the CA1 region of the AD specimen, while microglia in the healthy specimen have resting or ramified morphology by comparison. GFAP staining for astrogliosis was unremarkable for either specimen, although astrocytic plaques were found in the AD specimen.

**Fig. 11.**
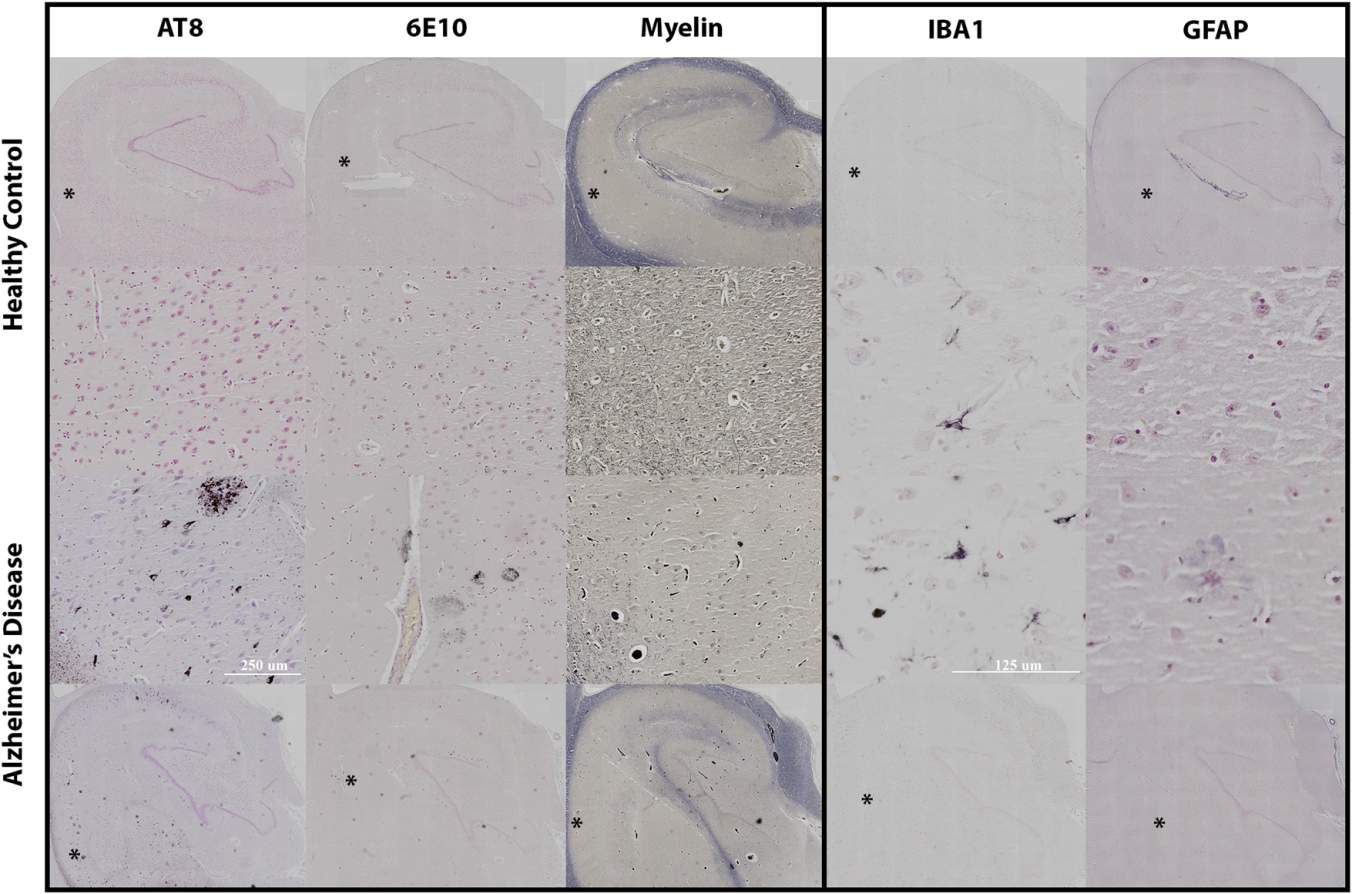
Histologic evaluations of AD pathology, myelin, and gliosis in representative healthy (top) and AD (bottom) specimens. The middle two rows are digitally magnified to show pathologic and cellular features within theCA1 subfield. The * indicates region of magnification, and scale bares are shown: the left three columns 250 microns and the right two columns 150 microns. Stains included: AT8 for tau tangle presence, 6E10 for beta amyloid plaques, the myelin by myelin specific protein, IBA1 for microglia, and GFAP for astrocytes.

## 4 Discussion

MRI microscopy of AD post-mortem human tissue creates the opportunity to directly compare quantitative MRI metrics with hallmark pathologic features that are defined by the neuropathology scoring systems to diagnose the disorder. This approach is essential for developing microstructural MRI markers of AD pathology, which is unknown in-vivo and often accompanied by comorbid pathologies. In the present study, high-quality dMRI and relaxometry maps were successfully acquired at high resolution and comprehensively compared to each other and with AD pathology. The general, major observations were that metrics of restriction and diffusivity explained the greatest portion variance across samples with different pathology according to PCA and that positive correlation between Braak score and diffusivity in the hippocampus and negative correlation with restriction metrics were the most prominent. This suggests that the loss of cellular structures and/or the increase of free water compartments are the dominating microstructural influence on the MRI metrics studied. The exception to this observation was the striking distinction between the PA metric associated with microscale anisotropy and FA, which is non-specific to macroscale or microscale anisotropy. PA was preferentially negatively correlated with AD pathology, which suggests that microscale anisotropy metrics could be uniquely suited as markers of cellular degradation in gray matter.

### 4.1 Restriction, diffusivity, and relaxivity: profile of neurodegeneration and plaques

Microstructural MRI is uniquely suited to probe the tissue environment of the brain by measuring altered relaxivity times and diffusivity values that are influenced by the presence or absence of macromolecules and cell membranes within a specified voxel. In the hippocampus, diffusivity metric values were found to have the greatest positive correlation with Braak score and the second most impactful grouping on PC1, while T1 and T2 relaxivity values were uncorrelated and were the least influential on PC1. The lack of T1 and T2 alterations is somewhat surprising given the prominent macromolecular and microstructural alterations in hippocampal tissue with AD pathology, but the observation is consistent with an unclear pattern of T1 and T2 findings over decades of research in humans and animal models (Tang et al., 2018). While the mean T1 values in the higher Braak stage groups are decreased compared to T1 of the lower stages (e.g.,Fig. 6, radar plot), there was high variability in the values and no strong correlation of T1 with Braak score (r_T1,Braak_= -0.21). Notably, it has been suggested that different pathologic features have opposing influence on T2 values, resulting in a small or absent net effect and that the heterogeneity of T2 values in the hippocampus may be more sensitive to AD pathology (Wearn et al., 2020). Additionally, it is well accepted that fixation reduces relaxometry timings for both T1 and T2 (Birkl et al., 2016;Dawe et al., 2009;Pfefferbaum et al., 2004;Raman et al., 2017) so that in-vivo relaxivity mapping could be sensitive to microstructure or macromolecular pathology that is not apparent in post-mortem studies. In contrast, the strong positive correlation of hippocampal diffusivity values—both DTI TR and DKI MSD—with Braak score suggests that reduction of barriers to water diffusion such as those that occur during neurodegeneration is detectable by diffusion MRI. This observation is also consistent with most AD studies using diffusion MRI (Clerx et al., 2012;Goveas et al., 2015) and the long established role for diffusion MRI in the Alzheimer’s Disease Neuroimaging Initiative standard MRI protocols (Jack et al., 2008). While increased diffusivity is associated with neurodegeneration in this study and others, it may not be as useful for early detection given that the likely AD feature related to this change is the loss of cell barriers and that diffusivity is known to be influenced by a wide range of pathology, including gliosis and edema, making it sensitive but not specific to pathology. Ultimately, these findings suggest that a more direct relationship exists between diffusion and tissue degeneration than for relaxometry, and that diffusivity is sensitive to AD-related neurodegenerative processes even if it may not be specific for AD pathology, especially at early stages.

More advanced microstructural MRI methods for both diffusion and relaxometry have promise to improve the sensitivity and specificity of MR markers to detect AD pathology. In the hippocampus, PC2 was greatly influenced by NG and MWF; however, neither was correlated with AD pathology. Previous rodent studies (Perez-Torres et al., 2014) have found increased magnetization transfer ratio (MTR), related to BPF (Stikov et al., 2011), in neural tissue with Aβ plaques. It is critical to note that the mouse models often do not exhibit degeneration and may have limited value in the development of markers. In the human specimens examined in our study, we found a negative correlation between BPF and plaque score, suggesting that the microstructural influence of degenerative processes outweighs any increase in BPF from the Aβ plaque load. Literature has indirectly suggested similarity between BPF and MWF where both have linear relationships to myelin volume fraction (West et al., 2018); however, in this study, there were no evident correlations or PCA groupings between MWF and BPF within the gray matter structures studied in this work, which have only modest myelin content. MWF may better characterize white matter pathology or tract degeneration in AD (Dean et al., 2017). In gray matter, it may still be possible for relaxometry-based techniques to positively identify increased macromolecular content associated with AD proteinopathy if more specific techniques that mitigate the influence of degeneration can be developed for AD specific pathology, for example by combination with diffusion.

Probing water restriction using non-Gaussian dMRI methods promises new tools for characterizing the microenvironment and could positively identify pathologic increases of small-pore features such as increased beading or varicosities (Benjamini et al., 2020;Ozarslan et al., 2013). Conversely, the present study found strong negative correlations between RTOP and MSK and AD pathology in the hippocampus and histologic staining in the same hippocampal tissue for representative samples (Fig. 11) qualitatively confirmed degeneration of neuronal structures and did not show prominent gliosis. While plaques and tangles were both present in the hippocampus, the lack of correlation between these and non-Gaussian metrics of restriction suggests that the predomination of degenerative processes occludes any selectivity of restriction metrics to plaque or tangle features. More sophisticated dMRI methods may ultimately provide positive markers of plaque and tangle pathology. For example, the diffusion encoding schemes used in the present study relied on gradient strength to modify diffusion weighting, but study of non-Gaussian diffusion behavior using time dependent encoding acquisitions would be explored for sensitivity to positive pathologic increases (Ozarslan et al., 2006;Xu et al., 2014). Along these lines, more advanced acquisition strategies using double diffusion encoding, q-space trajectory mapping, or combined diffusion-relaxometry strategies (Benjamini et al., 2016;Frank et al., 2020;Paulsen et al., 2015;Slator et al., 2021;Szczepankiewicz et al., 2019;Westin et al., 2016;Yang et al., 2018) could increase the selectivity of dMRI for positive detection of pathologic features.

Findings in the entorhinal cortex in the current study were less prominent than for the hippocampus but provided the only evidence for positive correspondence of restriction with AD pathology by associating plaque count in the EC with RTOP and MSK (Supplemental Fig. 2). The observation of increased restriction in the EC but not the hippocampus can potentially be explained by the progression of neurodegeneration in AD. Severe levels of neurodegeneration in the EC occur during earlier stages of AD than hippocampal degeneration (Braak & Braak, 1993). With complete neurodegeneration for all EC samples (i.e., both early and late Braak staged tissue), the relationship between water-restriction and Aβ plaques would be unveiled. Furthermore, the EC correlation for RTOP and MSK was exclusively with plaques, not tangles, and the plaque load was greatest for the EC (Table 1), suggesting that the plaque presence within the EC is main contributor to increased restriction. While little existing work has employed non-Gaussian dMRI to detect AD pathology, one previous in-vivo human study reported a negative finding for RTOP in which there was no correlation with PET-markers of AD (Aβ and tau) (Spotorno et al., 2022). These findings are generally consistent with the present study although they differ in RTOP findings, which may be explained by differences in spatial resolution, anatomical region of interest masks or by differences in image quality or dMRI metric values between in-vivo and ex-vivo studies.

### 4.2 Macroscale and microscale anisotropy can differentiate degeneration and tangles

Anisotropic metrics are of interest as AD markers for their potential sensitivity to early neurodegenerative processes in which the morphology and density of neurites and projection fibers change and may also detect organized gliosis or proteinopathy. Because these changes in AD affect both microscopic and macroscopic anisotropy, the DTI FA metric may not be able to specify early microscale degeneration, especially in gray matter regions where FA is generally low due to complex fiber architecture and any alteration of microscale anisotropy is likely to be masked. The MAP-MRI metric of PA is more specific to microscale anisotropy and far less influenced by fiber architecture in tissue (Avram et al., 2016;Hutchinson et al., 2018;Ozarslan et al., 2013). In the current study, FA and PA were not similar in PCA analysis and PA was found to have stronger correspondence with Braak score and NFT count than FA, although there was a stronger correlation between FA and Aβ plaques. The interpretation that PA and FA report distinct features of the tissue environment and are differentially affected by AD pathology was further supported by the histogram results of this study. While the hippocampal FA histogram distribution was unimodal, the PA distribution was bimodal with high PA values localized to regions with axonal pathways and lower values to the hippocampal layers predominated by neurites (Fig. 8). The more prominent reduction in PA was evident in earlier stages of pathology than the more modest FA changes and appeared to be more consistent with altered neurites than projection fibers. This was consistent with preservation of axonal pathways in myelin staining of the same sections (Fig. 11) and degraded microstructural features found by membrane staining and CSD FODs and tractography (Fig. 10). Furthermore, reactive astrocytes were absent in these specimens, suggesting that the underlying basis for PA reduction was driven by changes to neurites. Taken together, these findings suggest that microstructural anisotropy metrics such as PA may be more sensitive to earlier cellular alterations and may be able to distinguish between different types of cellular alterations. Previous studies investigating FA in both AD and cognitively impaired patients correspond with our findings of decreased FA in the hippocampal regions (Hong et al., 2010;Ryu et al., 2017). While transgenic mouse models reported increases in microstructural anisotropy due to protein increase and no neurodegeneration (Jensen et al., 2022), a human aging study found that not only did PA decrease but it was more sensitive to gray and white matter changes than FA (Bouhrara et al., 2023). However, there have been no existing studies that have directly evaluated microstructural anisotropy in human AD, although this is proposed as a major application for such dMRI techniques (Avram et al., 2016;Frank et al., 2020;Ozarslan et al., 2013;Yang et al., 2018). The present findings of selective microscale anisotropy reductions with Braak score and neurofibril tangle load without commensurate FA changes highlight the promise of metrics that can detect microstructural alterations in an anatomical region as geometrically complex as the hippocampus where FA is likely to be more influenced by local fiber geometry than by true anisotropy of cellular structures (Shepherd et al., 2007). Given the centrality of the hippocampus to AD pathology and clinical symptoms, microstructural anisotropy metrics such as PA may provide a new class of valuable new markers for AD pathology.

### 4.3 Limitations and future directions

This study has yielded promising findings regarding restriction, anisotropy, and diffusivity of AD post-mortem specimens, but it is imperative to acknowledge both evident and potential limitations due to the experimental design. This study included a total post-mortem dataset of 14 human temporal lobe specimens; however, only 11 were used for the radiologic-pathologic hippocampal correlation analysis, and 10 were used for the EC. While our descriptive statistics are informative, this dataset and our experimental design and number of samples is strong relative to most post-mortem MRI AD studies that range over 5–10 specimens (Apostolova et al., 2015;Benveniste et al., 1999;Meadowcroft et al., 2007;Zeineh et al., 2015), it should be acknowledged that our statistical power is limited by the number of cases within our study. Based on this consideration and the inherent multiple comparisons problem of evaluating many metrics, inferential statistical analysis was not appropriate. Instead, this study provides an important comparison of metrics by PCA and initial radiologic-pathologic evidence to inform future work. The results are presented in the context of related trends observed in prior studies and contribute to differentiating microstructural metrics for AD pathology detection.

Another important consideration in AD research is the effect of age and comorbidities, which were reported and used for specimen selection but not directly interrogated as factors of our analysis. The targeted study of age and specific comorbid pathologies will be an important goal of future work.

## 5 Conclusions

Advanced diffusion and relaxometry MRI microscopy were used to obtain high-quality maps of microstructural metrics in post-mortem temporal lobe specimens and revealed several novel observations related to AD microstructural MRI markers. PCA identified diffusion and restriction metrics as the main sources of variance across specimens, while more subtle contributions were noted for other metrics. Radiologic-pathologic correspondence was observed between Braak score and increased hippocampal diffusivity and decreased restriction, suggesting that degenerative processes that reduce cell membrane densities and increase free water drive the greatest changes detected by microstructural MRI metrics in this study. PCA revealed differences between PA and FA, and correlation analysis with histopathology suggests that PA is more sensitive to earlier AD changes than FA such that microstructural MRI may be an important area for future research and MRI marker development.

## Supplementary Material

Supplementary Material

## Data Availability

All code used for analysis is available here:https://github.com/Courtney-C/analysis-for-dMRI-in-PM-AD-paper.git. All metric maps for all specimens used in this study are available via the University of Arizona at this link:10.25422/azu.data.25246162.
