# Supplementary material for: Identification of diffusion, kurtosis, and propagator MRI markers of Alzheimer’s disease pathology in post-mortem human tissue
Source: Imaging Neurosci (Camb). 2024 May 8;2:imag-2-00164. doi: 10.1162/imag_a_00164 (PMC12272233; doi:10.1162/imag_a_00164)
Supplement: Supplementary Material [file imag_a_00164-supp.pdf]

Supplemental Material:

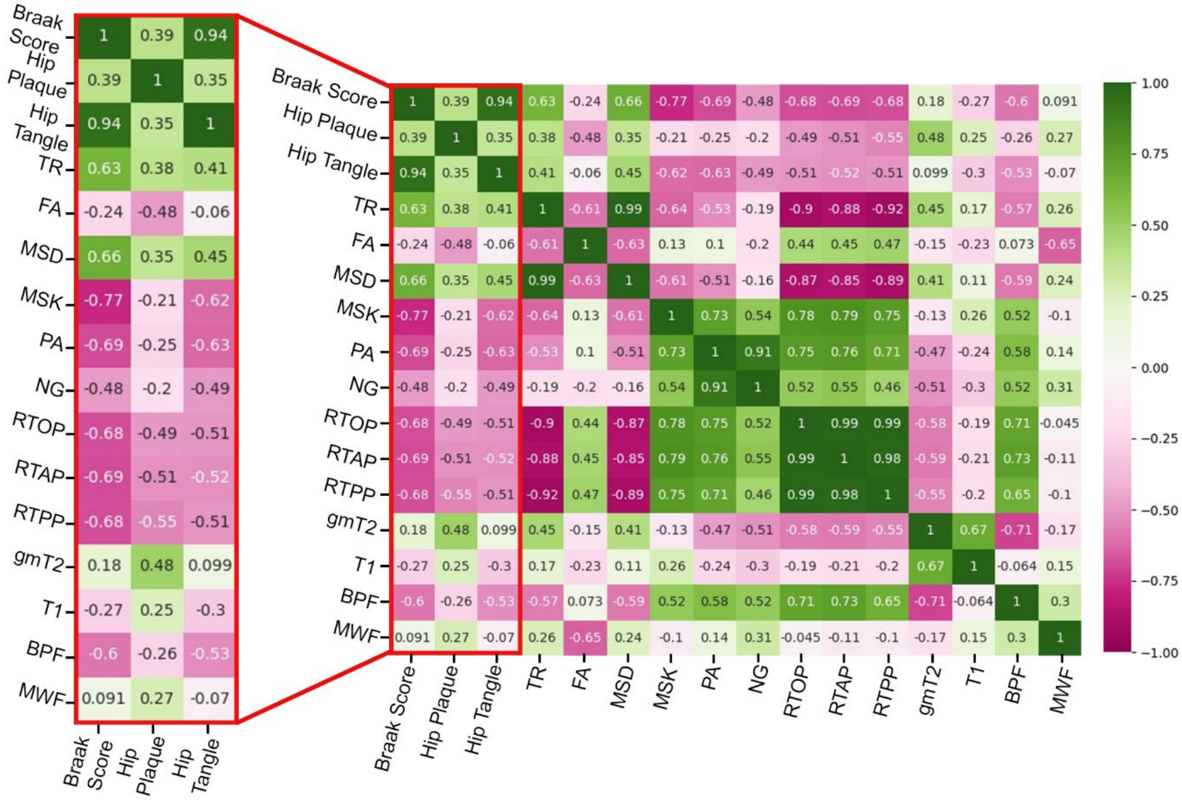

Supplemental Figure 1: Spearman's Correlation plot between microstructural MRI metrics and neuropathologic scores in the hippocampus. Negative correlations are indicated by pink, and positive correlations are indicated by green. Radiologic-pathologic correlations between MRI metrics and pathology scores are outlined with a red rectangle and enlarged to the left.

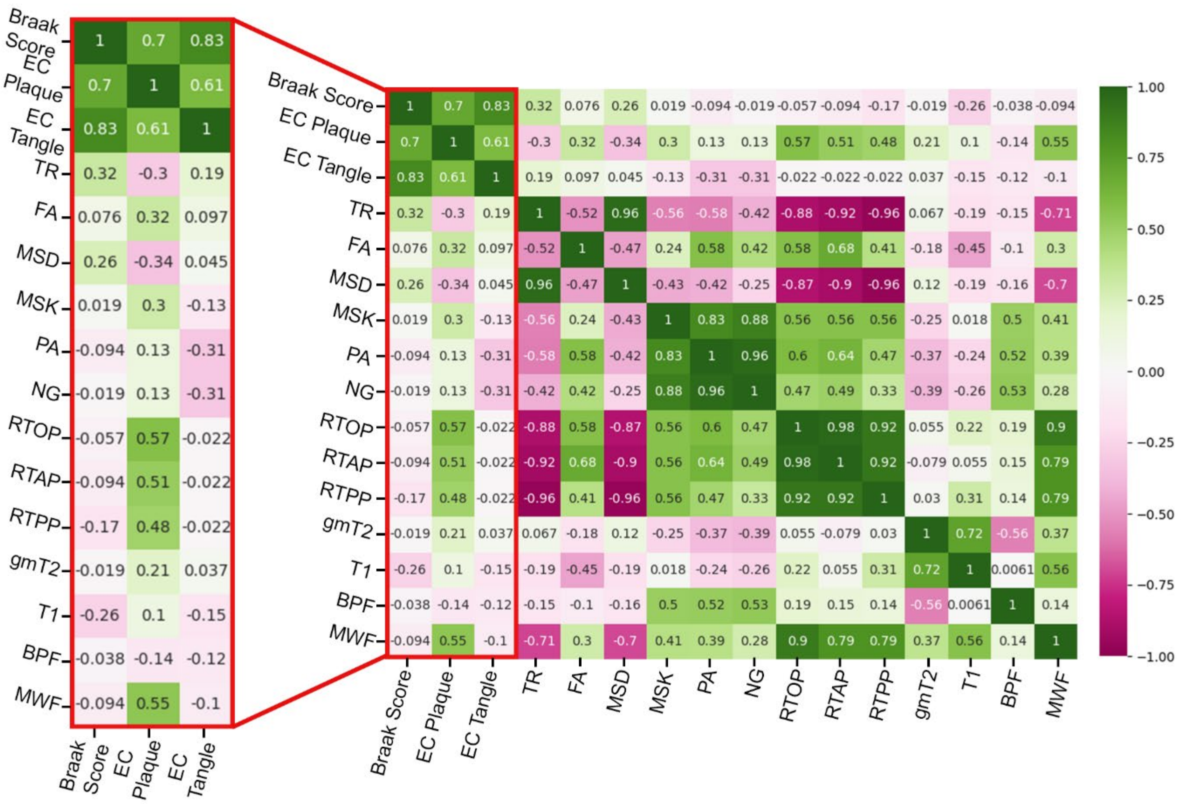

Supplemental Figure 2: Spearman's Correlation plot between microstructural MRI metrics and neuropathologic scores in the entorhinal cortex. Negative correlations are indicated by pink, and positive correlations are indicated by green. Radiologic-pathologic correlations between MRI metrics and pathology scores are outlined with a red rectangle and enlarged to the left.

Terms and abbreviations in order of first occurrence	
**Full Term**	**Abbreviation**
Alzheimer's Disease	AD
magnetic resonance imaging	MRI
positron emission tomography	PET
amyloid-b	Ab
neurofibril tangles	NFT
quantitative magnetization transfer	qMT
bound pool fraction	BPF
myelin water fraction	MWF
diffusion MRI	dMRI
diffusion tensor imaging	DTI
diffusion kurtosis imaging	DKI
mean apparent propagator	MAP
propagator anisotropy	PA
fractional anisotropy	FA
vascular dementia	VAD

progressive supranuclear palsy	PSP
hippocampal sclerosis	HS
dementia lacking distinctive histology	DLDH
motor neuron disease	MND
corticobasal degeneration	CBD
Huntington's disease	HD
multiple system atrophy	MSA
frontotemporal lobar dementia with TDP-43	FTLD-TDP
cerebral white matter rarefaction	CWMR
Lewy bodies	LBS
entorhinal cortex	EC
trace	TR
mean signal diffusion kurtosis imaging	MSDKI
mean signal diffusivity	MSD
mean signal kurtosis	MSK
return to origin probability	RTOP
quantitative magnetic transfer selective inversion recovery	qMT-SIR
high-resolution anatomical	HRA
region of interest	ROI
principal component analysis	PCA
confidence interval	CI
1,1'-Dioctadecyl-3,3,3',3'-Tetramethylindocarbocyanine Perchlorate	DiI
non-gaussianity	NG
stratum radiatum	SR
stratum oriens	SO
constrained spherical deconvolution	CSD
